# Bipolar multiplex families have an increased burden of common risk variants for psychiatric disorders

**DOI:** 10.1038/s41380-019-0558-2

**Published:** 2019-11-11

**Authors:** Till F. M. Andlauer, Jose Guzman-Parra, Fabian Streit, Jana Strohmaier, Maria José González, Susana Gil Flores, Francisco J. Cabaleiro Fabeiro, Francisco del Río Noriega, Fermin Perez Perez, Jesus Haro González, Guillermo Orozco Diaz, Yolanda de Diego-Otero, Berta Moreno-Küstner, Georg Auburger, Franziska Degenhardt, Stefanie Heilmann-Heimbach, Stefan Herms, Per Hoffmann, Josef Frank, Jerome C. Foo, Jens Treutlein, Stephanie H. Witt, Sven Cichon, Manolis Kogevinas, Bipolar Disorder Working Group of the Psychiatric Genomics Consortium, Eli A Stahl, Gerome Breen, Andreas J Forstner, Andrew McQuillin, Stephan Ripke, Vassily Trubetskoy, Manuel Mattheisen, Yunpeng Wang, Jonathan R I Coleman, Héléna A Gaspar, Christiaan A de Leeuw, Stacy Steinberg, Jennifer M Whitehead Pavlides, Maciej Trzaskowski, Tune H Pers, Peter A Holmans, Liam Abbott, Esben Agerbo, Huda Akil, Diego Albani, Ney Alliey-Rodriguez, Thomas D Als, Adebayo Anjorin, Verneri Antilla, Swapnil Awasthi, Judith A Badner, Marie Bækvad-Hansen, Jack D Barchas, Nicholas Bass, Michael Bauer, Richard Belliveau, Sarah E Bergen, Carsten Bøcker Pedersen, Erlend Bøen, Marco Boks, James Boocock, Monika Budde, William Bunney, Margit Burmeister, Jonas Bybjerg-Grauholm, William Byerley, Miquel Casas, Felecia Cerrato, Pablo Cervantes, Kimberly Chambert, Alexander W Charney, Danfeng Chen, Claire Churchhouse, Toni-Kim Clarke, William Coryell, David W Craig, Cristiana Cruceanu, Piotr M Czerski, Anders M Dale, Simone de Jong, Franziska Degenhardt, Jurgen Del-Favero, J Raymond DePaulo, Srdjan Djurovic, Amanda L Dobbyn, Ashley Dumont, Torbjørn Elvsåshagen, Valentina Escott-Price, Chun Chieh Fan, Sascha B Fischer, Matthew Flickinger, Tatiana M Foroud, Liz Forty, Josef Frank, Christine Fraser, Nelson B Freimer, Louise Frisén, Katrin Gade, Diane Gage, Julie Garnham, Claudia Giambartolomei, Marianne Giørtz Pedersen, Jaqueline Goldstein, Scott D Gordon, Katherine Gordon-Smith, Elaine K Green, Melissa J Green, Tiffany A Greenwood, Jakob Grove, Weihua Guan, José Guzman Parra, Marian L Hamshere, Martin Hautzinger, Urs Heilbronner, Stefan Herms, Maria Hipolito, Per Hoffmann, Dominic Holland, Laura Huckins, Stéphane Jamain, Jessica S Johnson, Anders Juréus, Radhika Kandaswamy, Robert Karlsson, James L Kennedy, Sarah Kittel-Schneider, James A Knowles, Manolis Kogevinas, Anna C Koller, Ralph Kupka, Catharina Lavebratt, Jacob Lawrence, William B Lawson, Markus Leber, Phil H Lee, Shawn E Levy, Jun Z Li, Chunyu Liu, Susanne Lucae, Anna Maaser, Donald J MacIntyre, Pamela B Mahon, Wolfgang Maier, Lina Martinsson, Steve McCarroll, Peter McGuffin, Melvin G McInnis, James D McKay, Helena Medeiros, Sarah E Medland, Fan Meng, Lili Milani, Grant W Montgomery, Derek W Morris, Thomas W Mühleisen, Niamh Mullins, Hoang Nguyen, Caroline M Nievergelt, Annelie Nordin Adolfsson, Evaristus A Nwulia, Claire O’Donovan, Loes M Olde Loohuis, Anil P S Ori, Lilijana Oruc, Urban Ösby, Roy H Perlis, Amy Perry, Andrea Pfennig, James B Potash, Shaun M Purcell, Eline J Regeer, Andreas Reif, Céline S Reinbold, John P Rice, Alexander L Richards, Fabio Rivas, Margarita Rivera, Panos Roussos, Douglas M Ruderfer, Euijung Ryu, Cristina Sánchez-Mora, Alan F Schatzberg, William A Scheftner, Nicholas J Schork, Cynthia Shannon Weickert, Tatyana Shehktman, Paul D Shilling, Engilbert Sigurdsson, Claire Slaney, Olav B Smeland, Janet L Sobell, Christine Søholm Hansen, Anne T Spijker, David St Clair, Michael Steffens, John S Strauss, Fabian Streit, Jana Strohmaier, Szabolcs Szelinger, Robert C Thompson, Thorgeir EThorgeirsson, Jens Treutlein, Helmut Vedde, Weiqing Wang, Stanley J Watson, Thomas W Weickert, Stephanie H Witt, Simon Xi, Wei Xu, Allan H Young, Peter Zandi, Peng Zhang, Sebastian Zollner, Rolf Adolfsson, Ingrid Agartz, Martin Alda, Lena Backlund, Bernhard T Baune, Frank Bellivier, Wade H Berrettini, Joanna M Biernacka, Douglas H R Blackwood, Michael Boehnke, Anders D Børglum, Aiden Corvin, Nicholas Craddock, Mark J Daly, Udo Dannlowski, Tõnu Esko, Bruno Etain, Mark Frye, Janice M Fullerton, Elliot S Gershon, Michael Gill, Fernando Goes, Maria Grigoroiu-Serbanescu, Joanna Hauser, David M Hougaard, Christina M Hultman, Ian Jones, Lisa A Jones, René S Kahn, George Kirov, Mikael Landén, Marion Leboyer, Cathryn M Lewis, Qingqin S Li, Jolanta Lissowska, Nicholas G Martin, Fermin Mayoral, Susan L McElroy, Andrew M McIntosh, Francis J McMahon, Ingrid Melle, Andres Metspalu, Philip B Mitchell, Gunnar Morken, Ole Mors, Preben Bo Mortensen, Bertram Müller-Myhsok, Richard M Myers, Benjamin M Neale, Vishwajit Nimgaonkar, Merete Nordentoft, Markus M Nöthen, Michael C O’Donovan, Ketil J Oedegaard, Michael J Owen, Sara A Paciga, Carlos Pato, Michele T Pato, Danielle Posthuma, Josep Antoni Ramos-Quiroga, Marta Ribasés, Marcella Rietschel, Guy A Rouleau, Martin Schalling, Peter R Schofield, Thomas G Schulze, Alessandro Serretti, Jordan W Smoller, Hreinn Stefansson, Kari Stefansson, Eystein Stordal, Patrick F Sullivan, Gustavo Turecki, Arne E Vaaler, Eduard Vieta, John B Vincent, Thomas Werge, John I Nurnberger, Naomi R Wray, Arianna Di Florio, Howard J Edenberg, Sven Cichon, Roel A Ophoff, Laura J Scott, Ole A Andreassen, John Kelsoe, Pamela Sklar, Major Depressive Disorder Working Group of the Psychiatric Genomics Consortium, Naomi R Wray, Stephan Ripke, Manuel Mattheisen, Maciej Trzaskowski, Enda M Byrne, Abdel Abdellaoui, Mark J Adams, Esben Agerbo, Tracy M Air, Till F M Andlauer, Silviu-Alin Bacanu, Marie Bækvad-Hansen, Aartjan T F Beekman, Tim B Bigdeli, Elisabeth B Binder, Julien Bryois, Henriette N Buttenschøn, Jonas Bybjerg-Grauholm, Na Cai, Enrique Castelao, Jane Hvarregaard Christensen, Toni-Kim Clarke, Jonathan R I Coleman, Lucía Colodro-Conde, Baptiste Couvy-Duchesne, Nick Craddock, Gregory E Crawford, Gail Davies, Ian J Deary, Franziska Degenhardt, Eske M Derks, Nese Direk, Conor V Dolan, Erin C Dunn, Thalia C Eley, Valentina Escott-Price, Farnush Farhadi Hassan Kiadeh, Hilary K Finucane, Jerome C Foo, Andreas J Forstner, Josef Frank, Héléna A Gaspar, Michael Gill, Fernando S Goes, Scott D Gordon, Jakob Grove, Lynsey S Hall, Christine Søholm Hansen, Thomas F Hansen, Stefan Herms, Ian B Hickie, Per Hoffmann, Georg Homuth, Carsten Horn, Jouke-Jan Hottenga, David M Hougaard, David M Howard, Marcus Ising, Rick Jansen, Ian Jones, Lisa A Jones, Eric Jorgenson, James A Knowles, Isaac S Kohane, Julia Kraft, Warren W. Kretzschmar, Zoltán Kutalik, Yihan Li, Penelope A Lind, Donald J MacIntyre, Dean F MacKinnon, Robert M Maier, Wolfgang Maier, Jonathan Marchini, Hamdi Mbarek, Patrick McGrath, Peter McGuffin, Sarah E Medland, Divya Mehta, Christel M Middeldorp, Evelin Mihailov, Yuri Milaneschi, Lili Milani, Francis M Mondimore, Grant W Montgomery, Sara Mostafavi, Niamh Mullins, Matthias Nauck, Bernard Ng, Michel G Nivard, Dale R Nyholt, Paul F O’Reilly, Hogni Oskarsson, Michael J Owen, Jodie N Painter, Carsten Bøcker Pedersen, Marianne Giørtz Pedersen, Roseann E Peterson, Erik Pettersson, Wouter J Peyrot, Giorgio Pistis, Danielle Posthuma, Jorge A Quiroz, Per Qvist, John P Rice, Brien P. Riley, Margarita Rivera, Saira Saeed Mirza, Robert Schoevers, Eva C Schulte, Ling Shen, Jianxin Shi, Stanley I Shyn, Engilbert Sigurdsson, Grant C B Sinnamon, Johannes H Smit, Daniel J Smith, Hreinn Stefansson, Stacy Steinberg, Fabian Streit, Jana Strohmaier, Katherine E Tansey, Henning Teismann, Alexander Teumer, Wesley Thompson, Pippa A Thomson, Thorgeir E Thorgeirsson, Matthew Traylor, Jens Treutlein, Vassily Trubetskoy, André G Uitterlinden, Daniel Umbricht, Sandra Van der Auwera, Albert M van Hemert, Alexander Viktorin, Peter M Visscher, Yunpeng Wang, Bradley T. Webb, Shantel Marie Weinsheimer, Jürgen Wellmann, Gonneke Willemsen, Stephanie H Witt, Yang Wu, Hualin S Xi, Jian Yang, Futao Zhang, Volker Arolt, Bernhard T Baune, Klaus Berger, Dorret I Boomsma, Sven Cichon, Udo Dannlowski, E J C de Geus, J Raymond DePaulo, Enrico Domenici, Katharina Domschke, Tõnu Esko, Hans J Grabe, Steven P Hamilton, Caroline Hayward, Andrew C Heath, Kenneth S Kendler, Stefan Kloiber, Glyn Lewis, Qingqin S Li, Susanne Lucae, Pamela A F Madden, Patrik K Magnusson, Nicholas G Martin, Andrew M McIntosh, Andres Metspalu, Ole Mors, Preben Bo Mortensen, Bertram Müller-Myhsok, Merete Nordentoft, Markus M Nöthen, Michael C O’Donovan, Sara A Paciga, Nancy L Pedersen, Brenda W J H Penninx, Roy H Perlis, David J Porteous, James B Potash, Martin Preisig, Marcella Rietschel, Catherine Schaefer, Thomas G Schulze, Jordan W Smoller, Kari Stefansson, Henning Tiemeier, Rudolf Uher, Henry Völzke, Myrna M Weissman, Thomas Werge, Cathryn M Lewis, Douglas F Levinson, Gerome Breen, Anders D Børglum, Patrick F Sullivan, Fabio Rivas, Fermín Mayoral, Bertram Müller-Myhsok, Andreas J. Forstner, Markus M. Nöthen, Marcella Rietschel

**Affiliations:** 1 Department of Translational Research in Psychiatry, Max Planck Institute of Psychiatry, Munich, Germany; 2 Department of Neurology, Klinikum rechts der Isar, School of Medicine, Technical University of Munich, Munich, Germany; 3 Department of Mental Health, University Regional Hospital of Málaga, Institute of Biomedicine of Málaga (IBIMA), Málaga, Spain; 4 Department of Genetic Epidemiology in Psychiatry, Central Institute of Mental Health, Medical Faculty Mannheim, Heidelberg University, Mannheim, Germany; 5 Department of Mental Health, Hospital of Puerto Real, Cádiz, Spain; 6 Department of Mental Health, University Hospital of Reina Sofia, Córdoba, Spain; 7 Department of Mental Health, Hospital of Jaén, Jaén, Spain; 8 Department of Mental Health, Hospital of Jerez de la Frontera, Jerez de la Frontera, Spain; 9 Department of Mental Health, Hospital Punta de Europa, Algeciras, Spain; 10 Unidad de Gestión Clínica del Dispositivo de Cuidados Críticos y Urgencias del Distrito Sanitario Málaga—Coin-Guadalhorce, Málaga, Spain; 11 Department of Personality, Assessment and Psychological Treatment, University of Malaga, Institute of Biomedicine of Málaga (IBIMA), Málaga, Spain; 12 Department of Neurology, Goethe University Medical School, Frankfurt am Main, Germany; 13 Institute of Human Genetics, University of Bonn, School of Medicine & University Hospital Bonn, Bonn, Germany; 14 Department of Biomedicine, University of Basel, Basel, Switzerland; 15 Institute of Neuroscience and Medicine (INM-1), Research Center Jülich, Jülich, Germany; 16 Barcelona Institute for Global Health (ISGlobal), Barcelona, Spain; 17 Munich Cluster for Systems Neurology (SyNergy), Munich, Germany; 18 Institute of Translational Medicine, University of Liverpool, Liverpool, UK; 19 Centre for Human Genetics, University of Marburg, Marburg, Germany; 20 Department of Psychiatry (UPK), University of Basel, Basel, Switzerland; 21 Department of Genetics and Genomic Sciences, Icahn School of Medicine at Mount Sinai, New York, NY USA; 22 Department of Psychiatry, Icahn School of Medicine at Mount Sinai, New York, NY USA; 23 Medical and Population Genetics, Broad Institute, Cambridge, MA USA; 24 MRC Social, Genetic and Developmental Psychiatry Centre, King’s College London, London, GB UK; 25 NIHR BRC for Mental Health, King’s College London, London, GB UK; 26 Human Genomics Research Group, Department of Biomedicine, University of Basel, Basel, CH Switzerland; 27 Department of Psychiatry (UPK), University of Basel, Basel, CH Switzerland; 28 Institute of Human Genetics, University of Bonn, School of Medicine & University Hospital Bonn, Bonn, DE Germany; 29 Department of Genomics, Life&Brain Center, University of Bonn, Bonn, DE Germany; 30 Institute of Medical Genetics and Pathology, University Hospital Basel, Basel, CH Switzerland; 31 Division of Psychiatry, University College London, London, GB UK; 32 Stanley Center for Psychiatric Research, Broad Institute, Cambridge, MA USA; 33 Department of Psychiatry and Psychotherapy, Charité - Universitätsmedizin, Berlin, DE Germany; 34 Analytic and Translational Genetics Unit, Massachusetts General Hospital, Boston, MA USA; 35 iSEQ, Center for Integrative Sequencing, Aarhus University, Aarhus, Denmark; 36 Department of Biomedicine - Human Genetics, Aarhus University, Aarhus, Denmark; 37 Department of Clinical Neuroscience, Centre for Psychiatry Research, Karolinska Institutet, Stockholm, SE Sweden; 38 Department of Psychiatry, Psychosomatics and Psychotherapy, Center of Mental Health, University Hospital Würzburg, Würzburg, DE Germany; 39 iPSYCH, The Lundbeck Foundation Initiative for Integrative Psychiatric Research, Risskov, Denmark; 40 Institute of Biological Psychiatry, Mental Health Centre Sct. Hans, Copenhagen, Denmark; 41 Institute of Clinical Medicine, University of Oslo, Oslo, NO Norway; 42 Department of Complex Trait Genetics, Center for Neurogenomics and Cognitive Research, Amsterdam Neuroscience, Vrije Universiteit Amsterdam, Amsterdam, The Netherlands; 43 deCODE Genetics / Amgen, Reykjavik, IS, Reykjavík, Iceland; 44 Queensland Brain Institute, The University of Queensland, Brisbane, QLD Australia; 45 Institute for Molecular Bioscience, The University of Queensland, Brisbane, QLD Australia; 46 Division of Endocrinology and Center for Basic and Translational Obesity Research, Boston Children’s Hospital, Boston, MA USA; 47 Medical Research Council Centre for Neuropsychiatric Genetics and Genomics, Division of Psychological Medicine and Clinical Neurosciences, Cardiff University, Cardiff, GB UK; 48 National Centre for Register-Based Research, Aarhus University, Aarhus, Denmark; 49 Centre for Integrated Register-based Research, Aarhus University, Aarhus, Denmark; 50 Molecular & Behavioral Neuroscience Institute, University of Michigan, Ann Arbor, MI USA; 51 NEUROSCIENCE, Istituto Di Ricerche Farmacologiche Mario Negri, Milano, Italy; 52 Department of Psychiatry and Behavioral Neuroscience, University of Chicago, Chicago, IL USA; 53 Psychiatry, Berkshire Healthcare NHS Foundation Trust, Bracknell, GB UK; 54 Psychiatry, Rush University Medical Center, Chicago, IL USA; 55 Center for Neonatal Screening, Department for Congenital Disorders, Statens Serum Institut, Copenhagen, Denmark; 56 Department of Psychiatry, Weill Cornell Medical College, New York, NY USA; 57 Department of Psychiatry and Psychotherapy, University Hospital Carl Gustav Carus, Technische Universität Dresden, Dresden, DE Germany; 58 Department of Medical Epidemiology and Biostatistics, Karolinska Institutet, Stockholm, SE Sweden; 59 Department of Psychiatric Research, Diakonhjemmet Hospital, Oslo, NO Norway; 60 Psychiatry, UMC Utrecht Hersencentrum Rudolf Magnus, Utrecht, The Netherlands; 61 Human Genetics, University of California Los Angeles, Los Angeles, CA USA; 62 Institute of Psychiatric Phenomics and Genomics (IPPG), University Hospital, LMU Munich, Munich, DE Germany; 63 Department of Psychiatry and Human Behavior, University of California, Irvine, Irvine, CA USA; 64 Molecular & Behavioral Neuroscience Institute and Department of Computational Medicine & Bioinformatics, University of Michigan, Ann Arbor, MI USA; 65 Psychiatry, University of California San Francisco, San Francisco, CA USA; 66 Instituto de Salud Carlos III, Biomedical Network Research Centre on Mental Health (CIBERSAM), Madrid, ES Spain; 67 Department of Psychiatry, Hospital Universitari Vall d´Hebron, Barcelona, ES Spain; 68 Department of Psychiatry and Forensic Medicine, Universitat Autònoma de Barcelona, Barcelona, ES Spain; 69 Psychiatric Genetics Unit, Group of Psychiatry Mental Health and Addictions, Vall d´Hebron Research Institut (VHIR), Universitat Autònoma de Barcelona, Barcelona, ES Spain; 70 Department of Psychiatry, Mood Disorders Program, McGill University Health Center, Montreal, QC Canada; 71 Division of Psychiatry, University of Edinburgh, Edinburgh, GB UK; 72 University of Iowa Hospitals and Clinics, Iowa City, IA USA; 73 Translational Genomics, USC, Phoenix, AZ USA; 74 Department of Translational Research in Psychiatry, Max Planck Institute of Psychiatry, Munich, DE Germany; 75 Department of Psychiatry, Laboratory of Psychiatric Genetics, Poznan University of Medical Sciences, Poznan, PL Poland; 76 Department of Neurosciences, University of California San Diego, La Jolla, CA USA; 77 Department of Radiology, University of California San Diego, La Jolla, CA USA; 78 Department of Psychiatry, University of California San Diego, La Jolla, CA USA; 79 Department of Cognitive Science, University of California San Diego, La Jolla, CA USA; 80 Applied Molecular Genomics Unit, VIB Department of Molecular Genetics, University of Antwerp, Antwerp, Belgium; 81 Department of Psychiatry and Behavioral Sciences, Johns Hopkins University School of Medicine, Baltimore, MD USA; 82 Department of Medical Genetics, Oslo University Hospital Ullevål, Oslo, NO Norway; 83 NORMENT, KG Jebsen Centre for Psychosis Research, Department of Clinical Science, University of Bergen, Bergen, NO Norway; 84 Department of Neurology, Oslo University Hospital, Oslo, NO Norway; 85 NORMENT, KG Jebsen Centre for Psychosis Research, Oslo University Hospital, Oslo, NO Norway; 86 Center for Statistical Genetics and Department of Biostatistics, University of Michigan, Ann Arbor, MI USA; 87 Department of Medical & Molecular Genetics, Indiana University, Indianapolis, IN USA; 88 Department of Genetic Epidemiology in Psychiatry, Central Institute of Mental Health, Medical Faculty Mannheim, Heidelberg University, Mannheim, DE Germany; 89 Center for Neurobehavioral Genetics, University of California Los Angeles, Los Angeles, CA USA; 90 Department of Molecular Medicine and Surgery, Karolinska Institutet and Center for Molecular Medicine, Karolinska University Hospital, Stockholm, SE Sweden; 91 Department of Clinical Neuroscience, Karolinska Institutet and Center for Molecular Medicine, Karolinska University Hospital, Stockholm, SE Sweden; 92 Child and Adolescent Psychiatry Research Center, Stockholm, SE Sweden; 93 Department of Psychiatry and Psychotherapy, University Medical Center Göttingen, Göttingen, DE Germany; 94 Department of Psychiatry, Dalhousie University, Halifax, NS Canada; 95 Genetics and Computational Biology, QIMR Berghofer Medical Research Institute, Brisbane, QLD Australia; 96 Department of Psychological Medicine, University of Worcester, Worcester, GB UK; 97 School of Biomedical and Healthcare Sciences, Plymouth University Peninsula Schools of Medicine and Dentistry, Plymouth, GB UK; 98 School of Psychiatry, University of New South Wales, Sydney, NSW Australia; 99 Bioinformatics Research Centre, Aarhus University, Aarhus, Denmark; 100 Biostatistics, University of Minnesota System, Minneapolis, MN USA; 101 Mental Health Department, University Regional Hospital, Biomedicine Institute (IBIMA), Málaga, ES Spain; 102 Department of Psychology, Eberhard Karls Universität Tübingen, Tubingen, DE Germany; 103 Department of Psychiatry and Behavioral Sciences, Howard University Hospital, Washington, DC USA; 104 Center for Multimodal Imaging and Genetics, University of California San Diego, La Jolla, CA USA; 105 Psychiatrie Translationnelle, Inserm, U955 Créteil, France; 106 Faculté de Médecine, Université Paris Est, Créteil, France; 107 Campbell Family Mental Health Research Institute, Centre for Addiction and Mental Health, Toronto, ON Canada; 108 Neurogenetics Section, Centre for Addiction and Mental Health, Toronto, ON Canada; 109 Department of Psychiatry, University of Toronto, Toronto, ON Canada; 110 Institute of Medical Sciences, University of Toronto, Toronto, ON Canada; 111 Department of Psychiatry, Psychosomatic Medicine and Psychotherapy, University Hospital Frankfurt, Frankfurt am Main, DE Germany; 112 Cell Biology, SUNY Downstate Medical Center College of Medicine, Brooklyn, NY USA; 113 Institute for Genomic Health, SUNY Downstate Medical Center College of Medicine, Brooklyn, NY USA; 114 ISGlobal, Barcelona, ES Spain; 115 Psychiatry, Altrecht, Utrecht, The Netherlands; 116 Psychiatry, GGZ inGeest, Amsterdam, The Netherlands; 117 Psychiatry, VU medisch centrum, Amsterdam, The Netherlands; 118 Psychiatry, North East London NHS Foundation Trust, Ilford, GB UK; 119 Clinic for Psychiatry and Psychotherapy, University Hospital Cologne, Cologne, DE Germany; 120 Psychiatric and Neurodevelopmental Genetics Unit, Massachusetts General Hospital, Boston, MA USA; 121 HudsonAlpha Institute for Biotechnology, Huntsville, AL USA; 122 Department of Human Genetics, University of Michigan, Ann Arbor, MI USA; 123 Psychiatry, University of Illinois at Chicago College of Medicine, Chicago, IL USA; 124 Max Planck Institute of Psychiatry, Munich, DE Germany; 125 Mental Health, NHS 24, Glasgow, GB UK; 126 Division of Psychiatry, Centre for Clinical Brain Sciences, University of Edinburgh, Edinburgh, GB UK; 127 Psychiatry, Brigham and Women’s Hospital, Boston, MA USA; 128 Department of Psychiatry and Psychotherapy, University of Bonn, Bonn, DE Germany; 129 Department of Genetics, Harvard Medical School, Boston, MA USA; 130 Department of Psychiatry, University of Michigan, Ann Arbor, MI USA; 131 Genetic Cancer Susceptibility Group, International Agency for Research on Cancer, Lyon, France; 132 Estonian Genome Center, University of Tartu, Tartu, EE Estonia; 133 Discipline of Biochemistry, Neuroimaging and Cognitive Genomics (NICOG) Centre, National University of Ireland, Galway, Galway, IE Ireland; 134 Neuropsychiatric Genetics Research Group, Dept of Psychiatry and Trinity Translational Medicine Institute, Trinity College Dublin, Dublin, IE Ireland; 135 Institute of Neuroscience and Medicine (INM-1), Research Centre Jülich, Jülich, DE Germany; 136 Research/Psychiatry, Veterans Affairs San Diego Healthcare System, San Diego, CA USA; 137 Department of Clinical Sciences, Psychiatry, Umeå University Medical Faculty, Umeå, SE Sweden; 138 Department of Clinical Psychiatry, Psychiatry Clinic, Clinical Center University of Sarajevo, Sarajevo, BA Bosnia and Herzegovina; 139 Department of Neurobiology, Care sciences, and Society, Karolinska Institutet and Center for Molecular Medicine, Karolinska University Hospital, Stockholm, SE Sweden; 140 Psychiatry, Harvard Medical School, Boston, MA USA; 141 Division of Clinical Research, Massachusetts General Hospital, Boston, MA USA; 142 Outpatient Clinic for Bipolar Disorder, Altrecht, Utrecht, The Netherlands; 143 Department of Psychiatry, Washington University in Saint Louis, Saint Louis, MO USA; 144 Department of Biochemistry and Molecular Biology II, Institute of Neurosciences, Center for Biomedical Research, University of Granada, Granada, ES Spain; 145 Department of Neuroscience, Icahn School of Medicine at Mount Sinai, New York, NY USA; 146 Medicine, Psychiatry, Biomedical Informatics, Vanderbilt University Medical Center, Nashville, TN USA; 147 Department of Health Sciences Research, Mayo Clinic, Rochester, MN USA; 148 Psychiatry and Behavioral Sciences, Stanford University School of Medicine, Stanford, CA USA; 149 Rush University Medical Center, Chicago, IL USA; 150 Scripps Translational Science Institute, La Jolla, CA USA; 151 Neuroscience Research Australia, Sydney, NSW Australia; 152 Faculty of Medicine, Department of Psychiatry, School of Health Sciences, University of Iceland, Reykjavik, IS Iceland; 153 Div Mental Health and Addiction, Oslo University Hospital, Oslo, NO Norway; 154 NORMENT, University of Oslo, Oslo, NO Norway; 155 Psychiatry and the Behavioral Sciences, University of Southern California, Los Angeles, CA USA; 156 Mood Disorders, PsyQ, Rotterdam, The Netherlands; 157 Institute for Medical Sciences, University of Aberdeen, Aberdeen, UK; 158 Research Division, Federal Institute for Drugs and Medical Devices (BfArM), Bonn, DE Germany; 159 Centre for Addiction and Mental Health, Toronto, ON Canada; 160 Neurogenomics, TGen, Los Angeles, AZ USA; 161 Psychiatry, Psychiatrisches Zentrum Nordbaden, Wiesloch, DE Germany; 162 Computational Sciences Center of Emphasis, Pfizer Global Research and Development, Cambridge, MA USA; 163 Department of Biostatistics, Princess Margaret Cancer Centre, Toronto, ON Canada; 164 Dalla Lana School of Public Health, University of Toronto, Toronto, ON Canada; 165 Psychological Medicine, Institute of Psychiatry, Psychology & Neuroscience, King’s College London, London, GB UK; 166 Department of Mental Health, Johns Hopkins University Bloomberg School of Public Health, Baltimore, MD USA; 167 Institute of Genetic Medicine, Johns Hopkins University School of Medicine, Baltimore, MD USA; 168 NORMENT, KG Jebsen Centre for Psychosis Research, Division of Mental Health and Addiction, Institute of Clinical Medicine and Diakonhjemmet Hospital, University of Oslo, Oslo, NO Norway; 169 National Institute of Mental Health, Klecany, CZ Czech Republic; 170 Discipline of Psychiatry, University of Adelaide, Adelaide, SA Australia; 171 Department of Psychiatry and Addiction Medicine, Assistance Publique - Hôpitaux de Paris, Paris, France; 172 Paris Bipolar and TRD Expert Centres, FondaMental Foundation, Paris, France; 173 UMR-S1144 Team 1: Biomarkers of relapse and therapeutic response in addiction and mood disorders, INSERM, Paris, France; 174 Psychiatry, Université Paris Diderot, Paris, France; 175 Psychiatry, University of Pennsylvania, Philadelphia, PA USA; 176 Department of Psychiatry, University of Münster, Münster, DE Germany; 177 Division of Endocrinology, Children’s Hospital Boston, Boston, MA USA; 178 Centre for Affective Disorders, Institute of Psychiatry, Psychology and Neuroscience, London, GB UK; 179 Department of Psychiatry & Psychology, Mayo Clinic, Rochester, MN USA; 180 School of Medical Sciences, University of New South Wales, Sydney, NSW Australia; 181 Department of Human Genetics, University of Chicago, Chicago, IL USA; 182 Biometric Psychiatric Genetics Research Unit, Alexandru Obregia Clinical Psychiatric Hospital, Bucharest, RO Romania; 183 Institute of Neuroscience and Physiology, University of Gothenburg, Gothenburg, SE Sweden; 184 INSERM, Paris, France; 185 Department of Medical & Molecular Genetics, King’s College London, London, GB UK; 186 Neuroscience Therapeutic Area, Janssen Research and Development, LLC, Titusville, NJ USA; 187 Cancer Epidemiology and Prevention, M. Sklodowska-Curie Cancer Center and Institute of Oncology, Warsaw, PL Poland; 188 School of Psychology, The University of Queensland, Brisbane, QLD Australia; 189 Research Institute, Lindner Center of HOPE, Mason, OH USA; 190 Centre for Cognitive Ageing and Cognitive Epidemiology, University of Edinburgh, Edinburgh, GB UK; 191 Human Genetics Branch, Intramural Research Program, National Institute of Mental Health, Bethesda, MD USA; 192 Division of Mental Health and Addiction, Oslo University Hospital, Oslo, NO Norway; 193 Division of Mental Health and Addiction, University of Oslo, Institute of Clinical Medicine, Oslo, NO Norway; 194 Institute of Molecular and Cell Biology, University of Tartu, Tartu, EE Estonia; 195 Mental Health, Faculty of Medicine and Health Sciences, Norwegian University of Science and Technology - NTNU, Trondheim, NO Norway; 196 Psychiatry, St Olavs University Hospital, Trondheim, NO Norway; 197 Psychosis Research Unit, Aarhus University Hospital, Risskov, Denmark; 198 Munich Cluster for Systems Neurology (SyNergy), Munich, DE Germany; 199 University of Liverpool, Liverpool, GB UK; 200 Psychiatry and Human Genetics, University of Pittsburgh, Pittsburgh, PA USA; 201 Mental Health Services in the Capital Region of Denmark, Mental Health Center Copenhagen, University of Copenhagen, Copenhagen, Denmark; 202 Division of Psychiatry, Haukeland Universitetssjukehus, Bergen, NO Norway; 203 Faculty of Medicine and Dentistry, University of Bergen, Bergen, NO Norway; 204 Human Genetics and Computational Biomedicine, Pfizer Global Research and Development, Groton, CT USA; 205 College of Medicine Institute for Genomic Health, SUNY Downstate Medical Center College of Medicine, Brooklyn, NY USA; 206 Department of Clinical Genetics, Amsterdam Neuroscience, Vrije Universiteit Medical Center, Amsterdam, The Netherlands; 207 Department of Neurology and Neurosurgery, McGill University, Faculty of Medicine, Montreal, QC Canada; 208 Montreal Neurological Institute and Hospital, Montreal, QC Canada; 209 Department of Biomedical and NeuroMotor Sciences, University of Bologna, Bologna, Italy; 210 Department of Psychiatry, Massachusetts General Hospital, Boston, MA USA; 211 Psychiatric and Neurodevelopmental Genetics Unit (PNGU), Massachusetts General Hospital, Boston, MA USA; 212 Faculty of Medicine, University of Iceland, Reykjavik, IS Iceland; 213 Department of Psychiatry, Hospital Namsos, Namsos, NO Norway; 214 Department of Neuroscience, Norges Teknisk Naturvitenskapelige Universitet Fakultet for naturvitenskap og teknologi, Trondheim, NO Norway; 215 Department of Genetics, University of North Carolina at Chapel Hill, Chapel Hill, NC USA; 216 Department of Psychiatry, University of North Carolina at Chapel Hill, Chapel Hill, NC USA; 217 Department of Psychiatry, McGill University, Montreal, QC Canada; 218 Dept of Psychiatry, Sankt Olavs Hospital Universitetssykehuset i Trondheim, Trondheim, NO Norway; 219 Clinical Institute of Neuroscience, Hospital Clinic, University of Barcelona, IDIBAPS, CIBERSAM, Barcelona, ES Spain; 220 Institute of Biological Psychiatry, MHC Sct. Hans, Mental Health Services Copenhagen, Roskilde, Denmark; 221 Department of Clinical Medicine, University of Copenhagen, Copenhagen, Denmark; 222 Psychiatry, Indiana University School of Medicine, Indianapolis, IN USA; 223 Biochemistry and Molecular Biology, Indiana University School of Medicine, Indianapolis, IN USA; 224 Institute for Molecular Bioscience, The University of Queensland, Brisbane, QLD Australia; 225 Queensland Brain Institute, The University of Queensland, Brisbane, QLD Australia; 226 Analytic and Translational Genetics Unit, Massachusetts General Hospital, Boston, MA USA; 227 Department of Psychiatry and Psychotherapy, Universitätsmedizin Berlin Campus Charité Mitte, Berlin, DE Germany; 228 Medical and Population Genetics, Broad Institute, Cambridge, MA USA; 229 Department of Psychiatry, Psychosomatics and Psychotherapy, University of Wurzburg, Wurzburg, DE Germany; 230 Centre for Psychiatry Research, Department of Clinical Neuroscience, Karolinska Institutet, Stockholm, SE Sweden; 231 Department of Biomedicine, Aarhus University, Aarhus, Denmark; 232 Dept of Biological Psychology & EMGO+ Institute for Health and Care Research, Vrije Universiteit Amsterdam, Amsterdam, The Netherlands; 233 Division of Psychiatry, University of Edinburgh, Edinburgh, GB UK; 234 Centre for Integrated Register-based Research, Aarhus University, Aarhus, Denmark; 235 National Centre for Register-Based Research, Aarhus University, Aarhus, Denmark; 236 iPSYCH, The Lundbeck Foundation Initiative for Integrative Psychiatric Research, Risskov, Denmark; 237 Discipline of Psychiatry, University of Adelaide, Adelaide, SA Australia; 238 Department of Translational Research in Psychiatry, Max Planck Institute of Psychiatry, Munich, DE Germany; 239 Department of Neurology, Klinikum rechts der Isar, Technical University of Munich, Munich, DE Germany; 240 Department of Psychiatry, Virginia Commonwealth University, Richmond, VA USA; 241 Center for Neonatal Screening, Department for Congenital Disorders, Statens Serum Institut, Copenhagen, Denmark; 242 Department of Psychiatry, Vrije Universiteit Medical Center and GGZ inGeest, Amsterdam, The Netherlands; 243 Virginia Institute for Psychiatric and Behavior Genetics, Richmond, VA USA; 244 Department of Psychiatry and Behavioral Sciences, Emory University School of Medicine, Atlanta, GA USA; 245 Department of Medical Epidemiology and Biostatistics, Karolinska Institutet, Stockholm, SE Sweden; 246 Department of Clinical Medicine, Translational Neuropsychiatry Unit, Aarhus University, Aarhus, Denmark; 247 iSEQ, Centre for Integrative Sequencing, Aarhus University, Aarhus, Denmark; 248 Human Genetics, Wellcome Trust Sanger Institute, Cambridge, GB UK; 249 Statistical genomics and systems genetics, European Bioinformatics Institute (EMBL-EBI), Cambridge, GB UK; 250 Department of Psychiatry, Lausanne University Hospital and University of Lausanne, Lausanne, CH Switzerland; 251 Social, Genetic and Developmental Psychiatry Centre, King’s College London, London, GB UK; 252 Genetics and Computational Biology, QIMR Berghofer Medical Research Institute, Brisbane, QLD Australia; 253 Centre for Advanced Imaging, The University of Queensland, Brisbane, QLD Australia; 254 Psychological Medicine, Cardiff University, Cardiff, GB UK; 255 Center for Genomic and Computational Biology, Duke University, Durham, NC USA; 256 Department of Pediatrics, Division of Medical Genetics, Duke University, Durham, NC USA; 257 Centre for Cognitive Ageing and Cognitive Epidemiology, University of Edinburgh, Edinburgh, GB UK; 258 Institute of Human Genetics, University of Bonn, School of Medicine & University Hospital Bonn, Bonn, DE Germany; 259 Epidemiology, Erasmus MC, Rotterdam, Zuid-Holland, The Netherlands; 260 Psychiatry, Dokuz Eylul University School Of Medicine, Izmir, TR Turkey; 261 Department of Psychiatry, Massachusetts General Hospital, Boston, MA USA; 262 Psychiatric and Neurodevelopmental Genetics Unit (PNGU), Massachusetts General Hospital, Boston, MA USA; 263 Stanley Center for Psychiatric Research, Broad Institute, Cambridge, MA USA; 264 Neuroscience and Mental Health, Cardiff University, Cardiff, GB UK; 265 Bioinformatics, University of British Columbia, Vancouver, BC Canada; 266 Department of Epidemiology, Harvard T.H. Chan School of Public Health, Boston, MA USA; 267 Department of Mathematics, Massachusetts Institute of Technology, Cambridge, MA USA; 268 Department of Genetic Epidemiology in Psychiatry, Central Institute of Mental Health, Medical Faculty Mannheim, Heidelberg University, Mannheim, Baden-Württemberg, DE Germany; 269 Department of Psychiatry (UPK), University of Basel, Basel, CH Switzerland; 270 Department of Biomedicine, University of Basel, Basel, CH Switzerland; 271 Centre for Human Genetics, University of Marburg, Marburg, DE Germany; 272 Department of Psychiatry, Trinity College Dublin, Dublin, IE Ireland; 273 Psychiatry & Behavioral Sciences, Johns Hopkins University, Baltimore, MD USA; 274 Bioinformatics Research Centre, Aarhus University, Aarhus, Denmark; 275 Institute of Genetic Medicine, Newcastle University, Newcastle upon Tyne, GB UK; 276 Danish Headache Centre, Department of Neurology, Rigshospitalet, Glostrup, Denmark; 277 Institute of Biological Psychiatry, Mental Health Center Sct, Hans, Mental Health Services Capital Region of Denmark, Copenhagen, Denmark; 278 iPSYCH, The Lundbeck Foundation Initiative for Psychiatric Research, Copenhagen, Denmark; 279 Brain and Mind Centre, University of Sydney, Sydney, NSW Australia; 280 Interfaculty Institute for Genetics and Functional Genomics, Department of Functional Genomics, University Medicine and Ernst Moritz Arndt University Greifswald, Greifswald, Mecklenburg-Vorpommern, DE Germany; 281 Roche Pharmaceutical Research and Early Development, Pharmaceutical Sciences, Roche Innovation Center Basel, F. Hoffmann-La Roche Ltd, Basel, CH Switzerland; 282 Max Planck Institute of Psychiatry, Munich, DE Germany; 283 MRC Centre for Neuropsychiatric Genetics and Genomics, Cardiff University, Cardiff, GB UK; 284 Department of Psychological Medicine, University of Worcester, Worcester, GB UK; 285 Division of Research, Kaiser Permanente Northern California, Oakland, CA USA; 286 Psychiatry & The Behavioral Sciences, University of Southern California, Los Angeles, CA USA; 287 Department of Biomedical Informatics, Harvard Medical School, Boston, MA USA; 288 Department of Medicine, Brigham and Women’s Hospital, Boston, MA USA; 289 Informatics Program, Boston Children’s Hospital, Boston, MA USA; 290 Wellcome Trust Centre for Human Genetics, University of Oxford, Oxford, GB UK; 291 Institute of Social and Preventive Medicine (IUMSP), Lausanne University Hospital and University of Lausanne, Lausanne, VD, CH Switzerland; 292 Swiss Institute of Bioinformatics, Lausanne, VD, CH Switzerland; 293 Division of Psychiatry, Centre for Clinical Brain Sciences, University of Edinburgh, Edinburgh, GB UK; 294 Mental Health, NHS 24, Glasgow, GB UK; 295 Department of Psychiatry and Psychotherapy, University of Bonn, Bonn, DE Germany; 296 Statistics, University of Oxford, Oxford, GB UK; 297 Psychiatry, Columbia University College of Physicians and Surgeons, New York, NY USA; 298 School of Psychology and Counseling, Queensland University of Technology, Brisbane, QLD Australia; 299 Child and Youth Mental Health Service, Children’s Health Queensland Hospital and Health Service, South Brisbane, QLD Australia; 300 Child Health Research Centre, University of Queensland, Brisbane, QLD Australia; 301 Estonian Genome Center, University of Tartu, Tartu, EE Estonia; 302 Medical Genetics, University of British Columbia, Vancouver, BC Canada; 303 Statistics, University of British Columbia, Vancouver, BC Canada; 304 DZHK (German Centre for Cardiovascular Research), Partner Site Greifswald, University Medicine, University Medicine Greifswald, Greifswald, Mecklenburg-Vorpommern, DE Germany; 305 Institute of Clinical Chemistry and Laboratory Medicine, University Medicine Greifswald, Greifswald, Mecklenburg-Vorpommern, DE Germany; 306 Institute of Health and Biomedical Innovation, Queensland University of Technology, Brisbane, QLD Australia; 307 Humus, Reykjavik, IS Iceland; 308 Virginia Institute for Psychiatric & Behavioral Genetics, Virginia Commonwealth University, Richmond, VA USA; 309 Clinical Genetics, Vrije Universiteit Medical Center, Amsterdam, The Netherlands; 310 Complex Trait Genetics, Vrije Universiteit Amsterdam, Amsterdam, The Netherlands; 311 Solid Biosciences, Boston, MA USA; 312 Department of Psychiatry, Washington University in Saint Louis School of Medicine, Saint Louis, MO USA; 313 Department of Biochemistry and Molecular Biology II, Institute of Neurosciences, Biomedical Research Center (CIBM), University of Granada, Granada, ES Spain; 314 Department of Psychiatry, University of Groningen, University Medical Center Groningen, Groningen, The Netherlands; 315 Department of Psychiatry and Psychotherapy, University Hospital, Ludwig Maximilian University Munich, Munich, DE Germany; 316 Institute of Psychiatric Phenomics and Genomics (IPPG), University Hospital, Ludwig Maximilian University Munich, Munich, DE Germany; 317 Division of Cancer Epidemiology and Genetics, National Cancer Institute, Bethesda, MD USA; 318 Behavioral Health Services, Kaiser Permanente Washington, Seattle, WA USA; 319 Faculty of Medicine, Department of Psychiatry, University of Iceland, Reykjavik, IS Iceland; 320 School of Medicine and Dentistry, James Cook University, Townsville, QLD Australia; 321 Institute of Health and Wellbeing, University of Glasgow, Glasgow, GB UK; 322 deCODE Genetics / Amgen, Reykjavik, IS Iceland; 323 College of Biomedical and Life Sciences, Cardiff University, Cardiff, GB UK; 324 Institute of Epidemiology and Social Medicine, University of Münster, Münster, Nordrhein-Westfalen, DE Germany; 325 Institute for Community Medicine, University Medicine Greifswald, Greifswald, Mecklenburg-Vorpommern, DE Germany; 326 Department of Psychiatry, University of California, San Diego, San Diego, CA USA; 327 KG Jebsen Centre for Psychosis Research, Norway Division of Mental Health and Addiction, Oslo University Hospital, Oslo, NO Norway; 328 Medical Genetics Section, CGEM, IGMM, University of Edinburgh, Edinburgh, GB UK; 329 Clinical Neurosciences, University of Cambridge, Cambridge, GB UK; 330 Internal Medicine, Erasmus MC, Rotterdam, Zuid-Holland, The Netherlands; 331 Roche Pharmaceutical Research and Early Development, Neuroscience, Ophthalmology and Rare Diseases Discovery & Translational Medicine Area, Roche Innovation Center Basel, F. Hoffmann-La Roche Ltd, Basel, CH Switzerland; 332 Department of Psychiatry and Psychotherapy, University Medicine Greifswald, Greifswald, Mecklenburg-Vorpommern, DE Germany; 333 Department of Psychiatry, Leiden University Medical Center, Leiden, The Netherlands; 334 Virginia Institute for Psychiatric & Behavioral Genetics, Virginia Commonwealth University, Richmond, VA USA; 335 Computational Sciences Center of Emphasis, Pfizer Global Research and Development, Cambridge, MA USA; 336 Institute for Molecular Bioscience; Queensland Brain Institute, The University of Queensland, Brisbane, QLD Australia; 337 Department of Psychiatry, University of Münster, Münster, Nordrhein-Westfalen, DE Germany; 338 Department of Psychiatry, University of Münster, Münster, DE Germany; 339 Department of Psychiatry, Melbourne Medical School, University of Melbourne, Melbourne, Australia; 340 Florey Institute for Neuroscience and Mental Health, University of Melbourne, Melbourne, Australia; 341 Institute of Medical Genetics and Pathology, University Hospital Basel, University of Basel, Basel, CH Switzerland; 342 Institute of Neuroscience and Medicine (INM-1), Research Center Juelich, Juelich, DE Germany; 343 Amsterdam Public Health Institute, Vrije Universiteit Medical Center, Amsterdam, The Netherlands; 344 Centre for Integrative Biology, Università degli Studi di Trento, Trento, Trentino-Alto Adige Italy; 345 Department of Psychiatry and Psychotherapy, Medical Center - University of Freiburg, Faculty of Medicine, University of Freiburg, Freiburg, DE Germany; 346 Center for NeuroModulation, Faculty of Medicine, University of Freiburg, Freiburg, DE Germany; 347 Psychiatry, Kaiser Permanente Northern California, San Francisco, CA USA; 348 Medical Research Council Human Genetics Unit, Institute of Genetics and Molecular Medicine, University of Edinburgh, Edinburgh, GB UK; 349 Department of Psychiatry, University of Toronto, Toronto, ON Canada; 350 Centre for Addiction and Mental Health, Toronto, ON Canada; 351 Division of Psychiatry, University College London, London, GB UK; 352 Neuroscience Therapeutic Area, Janssen Research and Development, LLC, Titusville, NJ USA; 353 Institute of Molecular and Cell Biology, University of Tartu, Tartu, EE Estonia; 354 Psychosis Research Unit, Aarhus University Hospital, Risskov, Aarhus, Denmark; 355 M5 (SyNergy), Munich, DE Germany; 356 University of Liverpool, Liverpool, GB UK; 357 Mental Health Center Copenhagen, Copenhagen Universtity Hospital, Copenhagen, Denmark; 358 Human Genetics and Computational Biomedicine, Pfizer Global Research and Development, Groton, CT USA; 359 Psychiatry, Harvard Medical School, Boston, MA USA; 360 Psychiatry, University of Iowa, Iowa City, IA USA; 361 Department of Psychiatry and Behavioral Sciences, Johns Hopkins University, Baltimore, MD USA; 362 Department of Psychiatry and Psychotherapy, University Medical Center Göttingen, Goettingen, Niedersachsen, DE Germany; 363 Human Genetics Branch, NIMH Division of Intramural Research Programs, Bethesda, MD USA; 364 Faculty of Medicine, University of Iceland, Reykjavik, IS Iceland; 365 Child and Adolescent Psychiatry, Erasmus MC, Rotterdam, Zuid-Holland, The Netherlands; 366 Psychiatry, Erasmus MC, Rotterdam, Zuid-Holland, The Netherlands; 367 Psychiatry, Dalhousie University, Halifax, NS Canada; 368 Division of Epidemiology, New York State Psychiatric Institute, New York, NY USA; 369 Department of Clinical Medicine, University of Copenhagen, Copenhagen, Denmark; 370 Department of Medical & Molecular Genetics, King’s College London, London, GB UK; 371 Psychiatry & Behavioral Sciences, Stanford University, Stanford, CA USA; 372 NIHR Maudsley Biomedical Research Centre, King’s College London, London, GB UK; 373 Genetics, University of North Carolina at Chapel Hill, Chapel Hill, NC USA; 374 Psychiatry, University of North Carolina at Chapel Hill, Chapel Hill, NC USA

**Keywords:** Bipolar disorder, Depression, Schizophrenia, Bipolar disorder, Depression

## Abstract

Multiplex families with a high prevalence of a psychiatric disorder are often examined to identify rare genetic variants with large effect sizes. In the present study, we analysed whether the risk for bipolar disorder (BD) in BD multiplex families is influenced by common genetic variants. Furthermore, we investigated whether this risk is conferred mainly by BD-specific risk variants or by variants also associated with the susceptibility to schizophrenia or major depression. In total, 395 individuals from 33 Andalusian BD multiplex families (166 BD, 78 major depressive disorder, 151 unaffected) as well as 438 subjects from an independent, BD case/control cohort (161 unrelated BD, 277 unrelated controls) were analysed. Polygenic risk scores (PRS) for BD, schizophrenia (SCZ), and major depression were calculated and compared between the cohorts. Both the familial BD cases and unaffected family members had higher PRS for all three psychiatric disorders than the independent controls, with BD and SCZ being significant after correction for multiple testing, suggesting a high baseline risk for several psychiatric disorders in the families. Moreover, familial BD cases showed significantly higher BD PRS than unaffected family members and unrelated BD cases. A plausible hypothesis is that, in multiplex families with a general increase in risk for psychiatric disease, BD development is attributable to a high burden of common variants that confer a specific risk for BD. The present analyses demonstrated that common genetic risk variants for psychiatric disorders are likely to contribute to the high incidence of affective psychiatric disorders in the multiplex families. However, the PRS explained only part of the observed phenotypic variance, and rare variants might have also contributed to disease development.

## Introduction

Bipolar disorder (BD), characterised by alternating episodes of mania and depression, has a lifetime prevalence of ~1% and is a substantial contributor to disability throughout the world [1]. Nevertheless, reliable data concerning the aetiology of BD remain scarce. The heritability of BD is estimated to be above 70% [2–4], thus demonstrating an important genetic component in the development of the disorder. Genome-wide association studies (GWAS) in case/control samples have reported that single-nucleotide polymorphisms (SNP) with minor allele frequencies (MAF) of ≥ 1% explain a substantial proportion of the genetic risk for BD [5–12]: the heritability explained by such common variants (i.e., the SNP heritability) is estimated to be 0.17–0.23 on a liability scale [12]. Common variants also make a substantial contribution to the development of schizophrenia (SCZ) and major depressive disorder (MDD) [13, 14]. These three psychiatric disorders have a shared genetic component, whereby relatives of patients with BD have, in addition to BD, an increased risk for MDD and SCZ [15]. In fact, GWAS have shown that many genetic risk variants are associated with all three disorders [16–21].

Besides common variants with small individual effects, rare variants with larger effects may also contribute to BD development [22, 23]. In theory, such rare variants should be enriched in families with a high prevalence of illness, termed multiplex families, in comparison to unrelated BD cases. However, it remains unclear whether and to what extent disease incidence in multiplex families is caused by rare variants, a high load of common variants, or a combination of both.

To elucidate the molecular genetic causes of BD, we established the Andalusian Bipolar Family (ABiF) study in 1997, which recruited BD multiplex families [24–26]. In the present analyses, we first investigated whether common genetic variants make a significant contribution to the occurrence of BD in ABiF families. Next, we examined whether BD development was attributable to (a) BD-specific risk variants, (b) variants conferring risk to all three disorders BD, MDD, and SCZ, or, (c) a combination of both. To this end, polygenic risk scores (PRS) based on GWAS of BD, MDD, and SCZ were calculated for and compared between ABiF family members and unrelated BD cases and unrelated controls from the same population. Because of the strong genetic correlation between BD, SCZ, and MDD, standard PRS for BD cannot distinguish between BD-specific risks and factors shared between these disorders. To differentiate between genetic risk shared across and specific to any of the three disorders, we calculated PRS of disorder-specific risk variants using genome-wide inferred statistics (GWIS) and PRS of shared risk variants. To evaluate the possibility that population or technical differences between cohorts biased the results on psychiatric PRS, PRS for late-onset Alzheimer’s disease (LOAD) and simulated PRS were analysed as negative controls. Assuming a polygenic model with a contribution of common risk variants, we expected increased psychiatric PRS in the ABiF family members compared to unrelated samples and increased psychiatric PRS in patients compared to controls.

## Materials and methods

### Sample description

The ABiF study recruited BD multiplex families in Andalusia, Spain [24–26]. The present analyses included 395 members of 33 ABiF families. Diagnoses were assigned by two trained clinicians according to the Diagnostic and Statistical Manual of Mental Disorders (DSM)-IV criteria using the best estimate approach [24]. Diagnoses comprised (Table 1 and Supplementary Table S1): BD, *n* = 166 (families (FAM)_BD_; BD type I (BD-I): *n* = 115; BD type II (BD-II): *n* = 41; not otherwise specified (NOS) BD: *n* = 10); MDD, *n* = 78 MDD (FAM_MDD_); no history of an affective disorder, *n* = 151 (FAM_unaffected_). Six unaffected individuals with a history of substance abuse were excluded from the analyses. Forty-four subjects married into the families and had no parent in the ABiF cohort (36 unaffected; 8 MDD). Furthermore, an independent, previously reported Spanish BD case/control (CC) sample was analysed. Here, BD cases (CC_BD_) were recruited from consecutive clinical admissions and BD was diagnosed, as in the ABiF families, using DSM-IV [9]; unrelated control individuals (CC_controls_) were recruited in the framework of the longitudinal European Community Respiratory Health Survey (ECRHS) study. Blood for genotyping was acquired at the ECRHS2 assessment in 2000–2001. After quality control (QC), the combined data set of both cohorts comprises data from 384 FAM (163 FAM_BD_, 73 FAM_MDD_, 142 FAM_unaffected_, and 6 FAM_unaffected_ with a history of substance abuse) and 438 CC subjects (161 unrelated BD cases; BD-I: *n* = 156; BD-II: *n* = 5) and 277 unrelated controls. Of the 161 CC_BD_ cases, 59 (36.6%) reported a family history of BD. However, in contrast to the data collection in the ABiF families, this information relied only on the self-report by the respective CC_BD_ patient, and was not validated via an interview of further family members. BD diagnoses were not available for the unrelated controls, but the self-reported prevalence of current depression in this cohort was 3.3% at the time of genotyping and the self-reported prevalence of lifetime depression was 14.4% at the follow-up 10 years after genotyping, indicating that the cohort is fairly representative of a typical population in regard to the prevalence of depression [27].

**Table 1:** Characteristics of 389 individuals from the 33 ABiF families, the 161 unrelated bipolar cases (CC_BD_), and the 277 unrelated controls (CC_controls_)

	FAM_BD_ (*n* = 166)	FAM_MDD_ (*n* = 78)	FAM_unaffected_ (*n* = 145)	CC_BD_ (*n* = 161)	CC_controls_ (*n* = 277)
Median age at interview (MAD)	40.5 (12.5)	44.5 (11.5)	44 (15)^d^	44 (11)^d^	43 (6)^b,^^c^ *Missing* *=* *3*
Median age at onset (MAD)	20 (5)^c^ *Missing* *=* *3*	26 (8)^a^ *Missing* *=* *1*		23 (6)^a^	
Female sex *n* (%)	103 (62.1)^b,d^	54 (69.2)^b,d^	51 (35.2)^a,c,d^	91 (56.5)^b^	132 (47.7)^a,b^
Married-in *n* (%)	0 (100.0)	8 (10.0)	36 (24.8)		
Educational level					
Primary school	118 (71.5)^c^	53 (68.0)	102 (70.3)^c^	88 (54.7)^a,b^	
Secondary school	39 (23.6)^c^	18 (23.1)^c^	36 (24.8)^c^	58 (36.0)^a,b^	
University degree	8 (4.9)	7 (9.0)	7 (4.8)	15 (9.3)	
	*Missing* *=* *1*				
Severe impairment during disorder	105 (65.6)^c^ *Missing* *=* *6*	4 (5.6)^a,c^ *Missing* *=* *6*		160 (99.4)^a^	
History of psychosis	110 (66.3)^c^	4 (5.1)^a,c^		159 (98.8)^a^	
History of suicide attempts	41 (24.7)^b^	2 (2.6)^a,c^	1 (0.7)^a,^^c^ *Missing* *=* *1*	40 (24.8)^b^	

Six unaffected individuals with a history of substance abuse were excluded from the analyses and are not shown in this table. Age and age at onset were analysed using Mann–Whitney *U*-tests; median and median absolute deviation (MAD) are shown. Categorical values were analysed using chi-squared (*χ*²) tests with two degrees (education) or one degree (other) of freedom; number (*n*) and percentage (%) of subjects are shown. *Missing*: number of individuals with missing data. All subjects passed QC in the FAM sample (numbers as shown in the table), but 11 family members were excluded during QC of the joint sample, therefore reported numbers differ slightly between comparisons. Note that the unaffected, married-in family members were excluded from analyses of the combined data set (FAM + CC sample) unless specified otherwise. Differences between the following groups were at least nominally significant (for details and *p*-values adjusted for multiple testing see Supplementary Table 1)

^a^Different from FAM_BD_

^b^Different from FAM_unaffected_

^c^Different from CC_BD_

^d^Different from CC_controls_

Note that, while all subjects passed QC in the family-only sample, 11 family members were excluded during QC of the joint sample because they showed significant differences in autosomal heterozygosity from the mean. Reported numbers of subjects thus differ slightly for different comparisons. The joint data set contained 35 unaffected, married-in family members who were excluded from analyses using the combined sample (unless specified otherwise). A detailed description of QC procedures is provided in the Supplementary Methods.

The study was approved by the respective local ethics committees (Comités de ética de la investigación provincial de Cádiz, Córdoba, Granada, Jaén and Málaga), and all participants provided written informed consent. For five adolescents (age 15–17 years), written informed consent was also obtained from the parents.

### Genotyping and imputation

Genome-wide genotyping of the FAM sample was carried out using the Illumina Infinium PsychArray BeadChip (PsychChip). QC and population substructure analyses were performed in PLINK v1.9 [28], as described in the Supplementary Methods. Genotyping and basic QC of the CC sample were conducted previously and are described elsewhere [9]. The study used two genotype data sets: Analyses of family members by themselves used variants genotyped on the PsychChip. For analyses on the combined FAM + CC sample, the genotype data of the CC data set were, for the variants genotyped in both samples, merged with the genotype data of the FAM sample. Both genotype data sets (family-only and combined) were imputed independently to the 1000 Genomes phase 3 reference panel using SHAPEIT and IMPUTE2 [29–31]. After imputation and post-imputation QC, the combined data set of both cohorts contained 6,862,461 variants with an INFO metric of ≥ 0.8 and a MAF of ≥ 1%. The imputed FAM data set without the CC subjects contained 8,628,089 variants.

### Calculation of polygenic risk scores

PRS were calculated in *R* v3.3 [32] using imputed genetic data. For each PRS, the effect sizes of variants below a selected *p*-value threshold, both obtained from large GWAS (training data), were multiplied by the imputed SNP dosage in the test data and then summed to produce a single PRS per threshold. Test statistics and alleles in the GWAS training data were flipped so that effect sizes were always positive. Thus, the PRS represent cumulative, additive risk. PRS were scaled to represent the relative risk load (minimum possible cumulative risk load = 0, maximum = 1). For each disorder, ten PRS based on different GWAS *p*-value thresholds (<5 × 10^−8^, <1 × 10^−7^, <1 × 10^−6^, <1 × 10^−5^, <1 × 10^−4^, <0.001, <0.01, <0.05, <0.1, <0.2) were calculated. The number of SNPs used for each PRS is shown in Supplementary Table S2. For additional details, see the Supplementary Methods.

For BD, MDD, and SCZ diagnoses, summary statistics of GWAS by the Psychiatric Genomics Consortium (PGC) were used as training data. For BD, the data freeze contained 20,352 cases and 31,358 controls [12]. As selected index patients from the ABiF families and the unrelated Spanish BD case/control data set were part of this BD GWAS, we recalculated summary statistics for this PGC GWAS without these Spanish samples, to prevent false-positive results caused by sample overlap between training and test samples. For MDD and SCZ, published data sets were used. These contained 130,664 cases and 330,470 controls for MDD [14] and 33,640 cases and 43,456 controls for SCZ [13]. There was no overlap between the subjects included in those GWAS and the ABiF and Spanish case/control samples. Variants with an INFO metric of < 0.6 in the GWAS summary statistics were removed.

*Shared* psychiatric PRS were generated using all variants showing an association at *p* < 0.05 in the GWAS of BD, SCZ, and MDD and for which effect sizes pointed in the same direction across studies. For this shared set of variants, *p*-values and effect sizes, used as weights in the PRS, were obtained using random-effects meta-analysis. PRS were then calculated using the meta-analysis summary statistics. We generated disorder-specific summary statistics to assess genetic risk unique to each disorder. To this end, genome-wide inferred statistics (GWIS) were calculated as explained in detail elsewhere [33]. For example, we calculated BD GWAS summary statistics corrected for the MDD GWAS results (BD-MDD). These BD-MDD GWIS results are similar to results obtained from a conditional analysis for BD corrected for MDD. They represent a genetic unique BD liability, which is estimated based on the heritability of BD and the coheritability of BD and MDD, both estimated using LD score regression [34]. As recommended for this method, variants with an INFO metric of <0.9 or >1.1 were removed. Disorder-specific PRS, e.g., BD-MDD PRS, were then calculated based on the corresponding GWIS summary statistics.

To confirm whether family members and BD cases had an increased PRS specifically for the tested psychiatric disorders but not because of population or technical differences between cohorts, PRS for late-onset Alzheimer’s disease (LOAD) were calculated as a negative control, based on a GWAS by the International Genomics of Alzheimer’s Project (IGAP) with 17,008 cases and 37,154 controls [35]. For additional details, see the Supplementary Methods. Furthermore, 10,000 simulated PRS for each of the ten *p*-value thresholds were calculated as negative controls. To this end, random variants from across the genome were drawn, using the same number of variants as for the BD PRS at each threshold and random effect sizes from the pool of all available BD, SCZ, and MDD effects. The code for simulating PRS is available at: https://gitlab.com/tillandlauer/abif-prs-analyses/.

### Statistical analysis

PRS analyses on binary variables (e.g., diagnoses and comparisons between cohorts) were conducted in *R* with the function *glmm.wald* of the package *GMMAT*, using a logistic mixed model, fitted by maximum likelihood using Nelder–Mead optimisation [36] to account for family structure. For logistic models, PRS underwent *Z*-score standardisation to generate comparable odds ratios (OR). Family structure was modelled as a random effect, with a genetic relationship matrix calculated on pruned genotype data in GEMMA [37].

Linear mixed models (LMMs) taking family structure into account were calculated using the function *polygenic* of the package *GenABEL* [38] for analyses of quantitative variables (anticipation and age at onset). In these analyses, test statistics, including 95% confidence intervals (CI), were calculated using bootstrapping (package *boot* [39, 40]) and *p*-values were validated using permutation analysis (10,000 permutations). In these permutation analyses, the null distribution of test statistics was empirically determined by repeating regression analyses 10,000 times with random sampling of phenotype data. To calculate a *p*-value, the number of tests were counted where a model with a random genotype-phenotype association showed the same or a more extreme *p*-value than the correct, non-randomised model and this number was divided by the total number of tests (10,000).

For each analysis of PRS, all ten PRS *p*-value thresholds were analysed. In analyses of the combined FAM and CC data set, sex was used as a covariate. In the analysis of FAM data alone, sex and age at the time of the interview were used as fixed effects covariates; whether an individual had married into the family was incorporated as a second random effect. Following the hypothesis that family members or subjects with a psychiatric diagnosis have increased PRS for psychiatric disorders, one-sided *p*-values were calculated for all PRS-based analyses. In all analyses, *p*-values below the significance threshold *α* = 0.05 were considered as nominally significant. Unless otherwise stated, this threshold was corrected for 10 × 6 = 60 tests using the Bonferroni method (*α* = 0.05/60 = 8.33 × 10^−4^). For further details, see the Supplementary Methods.

To determine whether population or technical differences might have influenced the observed effects independently of diagnosis groups, simulated PRS, generated as described above, were analysed. For each model, association statistics of the 10,000 simulated PRS were calculated for the ten *p*-value thresholds; the disorder PRS at the threshold showing the lowest mean association *p*-value was analysed further: The number of simulated PRS at this threshold that showed the same or a stronger association was counted and compared to the association of the disorder PRS. This count was used as the number of successes in a binomial test to estimate the probability of success. For computational efficiency, models were fitted using restricted maximum likelihood estimation and the average information optimisation algorithm for this analysis.

## Results

Figures 1 and 2 show the test statistics for the PRS with the training GWAS *p*-value threshold *p*_PRS_ that showed the strongest association per PRS type. Full results for all ten *p*_PRS_ per PRS type calculated using logistic mixed models are provided in Supplementary Figs. S1–S12 and in Supplementary Tables S3–S11.

**Fig. 1:**
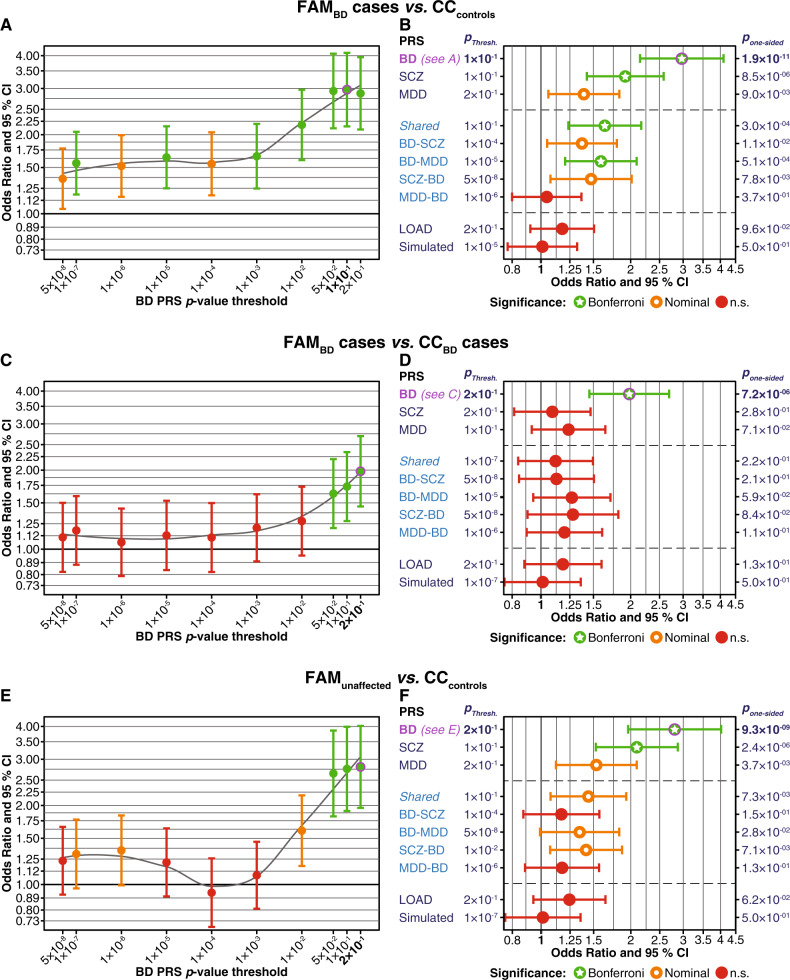
Comparison of PRS between FAM and CC samples. Married-in family members were excluded from these analyses. The plots show one-sided *p*-values, following the hypothesis that family members have higher PRS than individuals from the CC samples. All PRS have been normalised using *Z*-score standardisation. **a**, **b** Comparison of FAM_BD_ cases to CC_controls_. **a** FAM_BD_ cases had higher BD PRS across all ten *p*_PRS_ thresholds. The plot shows odds ratios (OR, *y*-axis, filled circles) and 95% confidence intervals (CI); *p*_PRS_ thresholds are shown on the *x*-axis. Results for each threshold are coloured by their degree of significance (one-sided *p*-values): red = not significant, orange = nominally significant, green = significant after Bonferroni correction for multiple testing (*α* = 0.05/60 = 0.00083). The top-associated PRS (*p*_PRS_ = 0.1) is indicated in bold font and was marked by a magenta circle (also in **b**). **b** For ten different PRS, this plot shows association statistics for the top-associated *p*_PRS_ thresholds. The *x*-axis shows ORs. BD, SCZ, MDD: Standard PRS using the respective PGC GWAS summary statistics. *Shared*: Shared psychiatric PRS (SNPs with BD, MDD, SCZ *p* < 0.05, random-effects meta-analysis). BD-SCZ, BD-MDD: BD-specific GWIS PRS corrected for SCZ and MDD, respectively. SCZ-BD and MDD-BD: GWIS PRS for SCZ and MDD, each corrected for BD. LOAD: PRS for late-onset Alzheimer’s disease. Simulated: Mean and CI of the 10,000 simulated PRS at the *p*_PRS_ with the lowest mean association *p*-value of all simulated PRS. The column to the left of the plot: *p*_PRS_ with the strongest association. Supplementary Fig. S2 shows plots for all *p*_PRS_. Column to the right: *p*_one-sided_ = one-sided *p*-value. For full association test statistics, see Supplementary Table S3. Bonferroni = significant after Bonferroni correction for multiple testing; nominal = nominally significant (*p* < 0.05); n.s. = not significant. **c**, **d** Comparison of FAM_BD_ cases and unrelated CC_BD_ cases. See Supplementary Fig. S3 and Table S4 for more detailed plots and full association test statistics. **e**, **f** Comparison of FAM_unaffected_ and CC_controls_. See Supplementary Fig. S4 and Table S5 for more detailed plots and full association test statistics

**Fig. 2:**
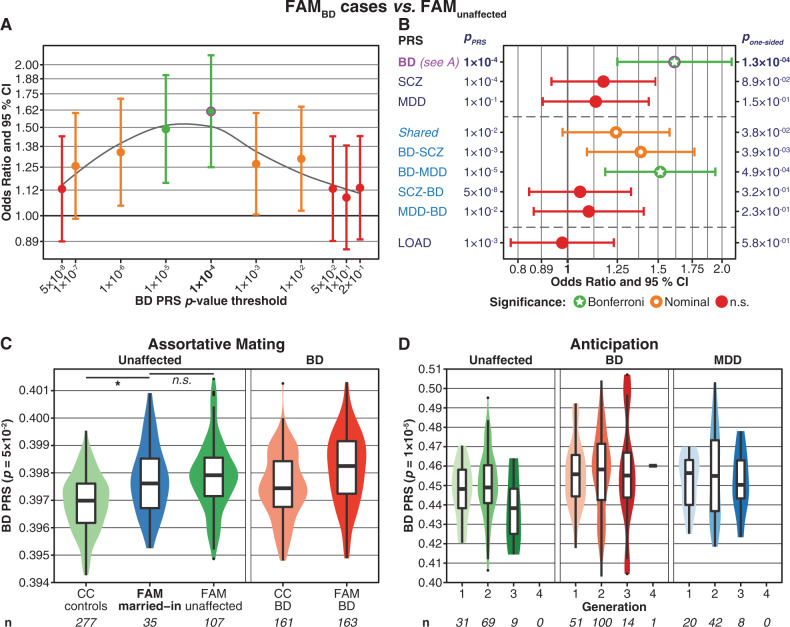
**a**, **b** Comparison of PRS between FAM_BD_ cases and FAM_unaffected_. The plots show one-sided *p*-values, following the hypothesis that BD cases have higher PRS than unaffected individuals. Further details of the plots are as described in the legend for Fig. 1. See Supplementary Fig. S5 and Table S6 for more detailed plots and full association test statistics. **c**, **d** Analyses of assortative mating (**c**) and anticipation (**d**). These plots were not adjusted for covariates; *n* = sample size. The *y*-axis shows the PRS values. **c:** Assortative mating. The plot shows violin- and boxplots of the BD PRS (*p*_PRS_ = 0.05), comparing unaffected, married-in individuals with no parent among the ABiF families to other FAM and CC subjects. At *p*_PRS_ = 0.05, married-in family members showed the highest BD PRS compared to CC_controls_ (*p* = 6.5 × 10^−5^, Supplementary Fig. S6A and Table S7). The BD PRS of married-in individuals was not significantly higher than the PRS of FAM_unaffected_ at any *p*_PRS_ (*p* ≥ 0.167, Supplementary Fig. S6B and Table S7). Covariate used: sex. One-sided *p*-values were calculated, following the hypothesis that married-in individuals have higher PRS than other unaffected subjects. Note that, in the context of assortative mating, the boxplots of affected BD cases are displayed for reference only and have not been included in the analysis. **d** Anticipation: the BD PRS did not increase across generations. The plot shows violin- and boxplots of the BD PRS (*p*_PRS_ = 1 × 10^−5^) across different generations of the FAM sample for the three diagnosis groups. At *p*_PRS_ = 1 × 10^−5^, the association of the BD PRS with generation was strongest but not significant (*p* = 0.45; Supplementary Fig. S7A and Table S8). Married-in family members were excluded from this analysis. Covariates used: sex, age at the interview, diagnostic group. One-sided *p*-values were calculated, following the hypothesis that the PRS increase across generations

### FAM_BD_ cases had higher psychiatric PRS than controls from the general population

On average, familial FAM_BD_ cases had higher BD PRS than unrelated CC_controls_ across the *p*_PRS_ thresholds (Fig. 1a, b; Supplementary Figs. S1 and S2 and Supplementary Table S3). The most substantial support for an increased BD PRS was found with the threshold *p*_PRS_ = 0.1 (OR = 2.97, one-sided *p* = 1.9 × 10^−11^). FAM_BD_ cases also had significantly higher SCZ PRS than CC_controls_; the increase of the MDD PRS was nominally significant (Fig. 1b).

*Shared* PRS generated from the variants associated jointly with BD, SCZ, and MDD were significantly increased at *p*_PRS_ ≥ 0.01 in FAM_BD_ cases compared to CC_controls_. The GWIS BD-MDD PRS—the BD PRS corrected for associations shared with MDD—were significantly increased in FAM_BD_ cases compared to CC_controls_. All other disorder-specific GWIS PRS were not significantly higher in FAM_BD_ cases after correction for multiple testing.

No significant increase was found for the negative-control PRS for late-onset Alzheimer’s disease (LOAD) and associations of the PRS for BD and SCZ and of the *Shared* PRS were significantly stronger than simulated PRS in FAM_BD_ compared to CC_controls_ (Table 2).

**Table 2:** The psychiatric disorder PRS can distinguish better between groups than simulated PRS

Group	Disorder PRS	Simulated PRS min. *p*_PRS_	N simulated PRS with *p* ≤ *p* of disorder PRS	Prob. of success	95% CI
FAM_BD_ vs. CC_controls_	BD	1 × 10^−5^	0	**<1** **×** **10**^**−4**^	**0–0**
	SCZ	1 × 10^−5^	0	**<1** **×** **10**^**−4**^	**0–0**
	MDD	1 × 10^−5^	91	0.0091	0.007–0.011
	*Shared*	1 × 10^−5^	2	**0.0002**	**0–0.001**
FAM_BD_ vs. CC_BD_	BD	1 × 10^−7^	0	**<1** **×** **10**^**−4**^	**0–0**
	SCZ	1 × 10^−7^	2858	0.2858	0.277–0.295
	MDD	1 × 10^−7^	744	0.0744	0.069–0.080
	*Shared*	1 × 10^−7^	2229	0.2229	0.215–0.231
FAM_unaffected_ vs. CC_controls_	BD	1 × 10^−7^	0	**<1** **×** **10**^**−4**^	**0–0**
	SCZ	1 × 10^−7^	0	**<1** **×** **10**^**−4**^	**0–0**
	MDD	1 × 10^−7^	37	0.0037	0.003–0.005
	*Shared*	1 × 10^−7^	74	0.0074	0.006–0.009
FAM_MDD_ vs. CC_controls_	BD	2 × 10^−1^	0	**<1** **×** **10**^**−4**^	**0–0**
	SCZ	2 × 10^−1^	409	0.0409	0.037–0.045
	MDD	2 × 10^−1^	2	**0.0002**	**0–0.001**
	*Shared*	2 × 10^−1^	125	0.0125	0.010–0.015

In binomial tests with 10,000 trials, the number of successes was the number of simulated PRS that showed the same or a stronger association than the disorder PRS (one-sided *p*-values). The 10,000 simulated PRS with ten *p*-value thresholds each were calculated by drawing random variants from across the genome, using the same number of variants as for the BD PRS at each threshold and random effect sizes from the pool of all available BD, SCZ, and MDD effects. For the present test, the *p*_PRS_ of the simulated PRS showing the lowest mean association *p*-value was chosen. *Prob*. = binomial test probability estimate of success; *CI* = confidence interval of the probability estimate, both calculated using the *R* package *binom* (method: *exact*). Significance threshold: 0.05/16 = 0.003125, comparisons surpassing this threshold are shown in bold font

### FAM_BD_ cases had higher BD PRS than unrelated CC_BD_ cases

The BD PRS was significantly higher in FAM_BD_ than in CC_BD_ cases at *p*_PRS_ ≥ 0.05, but no other type of PRS was increased in FAM_BD_ compared to CC_BD_ cases (Fig. 1c, d). The association of the BD PRS was significantly stronger than simulated PRS (Table 2).

### Unaffected family members showed higher psychiatric PRS than CC controls

In the comparison of FAM_unaffected_ to CC_controls_, PRS for BD and SCZ were significantly higher in unaffected family members (Fig. 1e, f). The increases of the MDD, *Shared*, BD-MDD, and SCZ-BD GWIS PRS were nominally significant. The associations of BD and SCZ PRS were significantly stronger than the associations of simulated PRS (Table 2).

### FAM_BD_ cases had an increased PRS specifically for BD

In comparison to FAM_unaffected_, the BD PRS and the BD-MDD disorder-specific PRS were significantly higher in FAM_BD_ (Fig. 2a, b). The *Shared* and the BD-SCZ PRS were increased at nominal significance.

### Effects of assortative mating on BD PRS in family members

Eight of the 44 individuals who had married into the families had a diagnosis of MDD and none of BD (Table 1). While the unaffected married-in individuals had higher BD PRS than CC_controls_ (*p* = 6.5 × 10^−5^), their BD PRS was not higher than the PRS of other FAM_unaffected_ (Fig. 2c; Supplementary Fig. S6 and Supplementary Table S7). We also examined possible anticipation of BD in the families: neither did the BD PRS increase significantly over generations nor did the age at onset decrease over time (Fig. 2d; Supplementary Fig. S7 and Supplementary Table S8).

### FAM_MDD_ cases had higher psychiatric PRS than CC_controls_

In comparison to CC_controls_, FAM_MDD_ cases had significantly higher BD and MDD PRS, increases of the *Shared* and SCZ PRS were nominally significant (Supplementary Figs. S8 and S9 and Supplementary Table S9). Both the BD and MDD PRS were increased at nominal significance when comparing FAM_MDD_ to FAM_unaffected_ (Supplementary Fig. S10 and Supplementary Table S10). Notably, in both comparisons, FAM_MDD_ showed a nominal increase in SCZ-MDD PRS, but not in SCZ-BD PRS.

## Discussion

Genome-wide association studies in large samples of unrelated patients and controls have unravelled the polygenic nature of BD, i.e., many common variants, each with a small effect size, contribute to BD. It has also been consistently shown that BD, MDD, and SCZ share many risk-conferring variants. The aim of the present study was to investigate whether common variants also contribute to BD in families with a high density of the disorder and if so, whether these variants are specific to BD.

We found that, compared to CC_controls_, unrelated subjects from the general population unscreened for BD, affected and unaffected ABiF family members had an elevated genetic risk for the tested psychiatric disorders, mainly for BD but also for SCZ. FAM_BD_ cases were characterised by a particularly high load of BD-specific risk variants: The strongest association observed across all comparisons was the increase of the BD PRS in FAM_BD_ compared to CC_controls_. In addition, the BD but not the SCZ and MDD PRS of FAM_BD_ were significantly higher than the PRS of unrelated CC_BD_ cases and unaffected family members. Together with the disorder-specific GWIS PRS, these results support the major contribution of BD-associated variants to the high density of the disorder in the investigated families.

An increased polygenic psychiatric risk has also been described in other studies of BD multiplex families [41–43]. However, the scope and results of these studies differed from the present study to some extent: Fullerton et al. [41] described an increased BD PRS in affected family members compared to unrelated controls and, when selecting families with a high polygenic BD risk load, also to unaffected family members. They constructed PRS only based on a small set of 32 SNPs from an older GWAS [10], and no other PRS were investigated. De Jong et al. [43] focused their analyses in a large Brazilian family with BD and MDD on assortative mating and anticipation and found BD and SCZ PRS to be increased at nominal significance in affected compared to unaffected members. In a large Swedish pedigree with mainly BD but also some SCZ cases, Szatkiewicz et al. [42] reported increased SCZ PRS in affected family members compared to family-level and population controls, as well as BD PRS increased at nominal significance in affected family members compared to family controls. However, no differences were observed between unaffected family members and population controls. Of note, none of these studies investigated differences in PRS between families and unrelated BD cases.

Compared to the CC_BD_ in our study, FAM_BD_ displayed, apart from an earlier age at onset, signs of a less severe clinical picture, i.e., less frequent impairment and less psychosis. This could be explained by the fact that CC_BD_ cases were almost all BD-I patients recruited from consecutive admissions to a hospital, while most of the FAM_BD_ cases were reached through other family members in the context of the study. Apart from this, the FAM_BD_ did not display any striking differences in clinical features compared to the CC_BD_. Thus, we consider it likely that the increased PRS in the FAM_BD_ is linked to the familial aggregation and not to clinical characteristics.

It appears striking that none of the ABiF family members have been diagnosed with SCZ. However, this can most likely be attributed to ascertainment bias as the recruitment strategy focused on BD multiplex families. With respect to this lack of SCZ diagnoses in the ABiF families, it is of interest that the family members showed not only an increased BD PRS but also increased SCZ and *Shared* PRS compared to unrelated controls. This increase could be an indirect consequence of the genetic correlation between BD and SCZ [14, 16, 18–21]. Furthermore, affected family members also had higher *Shared* PRS than CC_controls_. Of the psychiatric disorder GWAS data sets (i.e., SCZ, BD, and MDD) used in the present analysis, the SCZ GWAS both identified the largest amount of risk loci (108, 30, and 44, respectively) and the corresponding PRS explained the highest amount of case/control variance (7%, 4%, and 2% on a liability scale, respectively) [12–14]. Taking this and the genetic correlations between the disorders into account, the SCZ PRS might have included more cross-disorder signals with smaller effects than the PRS of BD and MDD. If family members had an increased *Shared* risk burden, this cross-disorder risk might have rendered them vulnerable to psychiatric disorders in general, with the high BD PRS then shaping the final BD diagnosis outcome. Of note, the analyses of FAM_MDD_ cases are discussed in the Supplementary Data.

Our study furthermore indicates that assortative mating may have contributed to the increased BD PRS in the ABiF families: in their study, de Jong et al. [43] found no increased PRS in married-in subjects, but an increase of polygenic risk and a decrease in age at onset over generations. We observed that individuals who married into the ABiF families had higher BD PRS than CC_controls_, and their BD risk load was similar to other FAM_unaffected_. At the time of the interview, none of the married-in family members had a diagnosis of BD. Nevertheless, their increased BD PRS suggests that assortative mating may have occurred. Unaffected individuals with an above average BD PRS may display sub-threshold characteristics of BD, such as a broader range of emotions [44–46]. Consistent with the observation that married-in subjects did not have higher BD PRS than the other FAM_unaffected_, no increase in BD PRS was found across generations. However, assortative mating may have contributed to the establishment and maintenance of a high genetic risk load for BD in these families. Furthermore, assortative mating may have already occurred in previous generations, for which no DNA was available. Of note, DNA was not available for all ABiF family members of the current generations, limiting the scope of the analysis of assortative mating.

Although both the FAM and CC samples were recruited in Spain [9], minor population differences may have influenced the present results. Even if such minor differences existed, it is unlikely that they caused the highly significant associations observed for the psychiatric PRS, given that the pairwise genetic relationship matrix was used as a random effect in the association analyses. Additionally, results from three further analyses support our assumption that systematic differences between the genotype data of FAM, CC_controls_, and CC_BD_ samples did not distort our findings: First, we did not find significant differences between the cohorts in a population substructure analysis (see Supplementary Fig. S11 and Supplementary Methods). Second, PRS for LOAD were not significantly increased in family members in any analysis. Since LOAD shows no genetic correlation with BD, MDD, or SCZ [14, 47, 48], this result further supports the specificity of our analyses. Third, when a psychiatric disorder PRS was significantly increased in family members, this association was stronger than for simulated PRS. While these findings cannot entirely exclude the influence of unknown confounders on our results, we consider them as strong evidence that the high psychiatric PRS observed in family members compared to controls cannot be attributed to population or technical differences between the cohorts.

The lower a *p*_PRS_ threshold in the GWAS training data, the fewer SNPs were included in the calculation of the corresponding PRS. In most cases, significant differences between groups were not observed for these low *p*_PRS_ but the higher thresholds based on thousands of variants. This is commonly observed and in line with the polygenic nature of psychiatric disorders as complex disorders, with genome-wide significant SNPs only accounting for a small share of the polygenic signal. The training GWAS used for BD, SCZ, and MDD, the largest available for these phenotypes, differ in the number of included subjects, their statistical power, and the number of identified signals. Therefore, the derived PRS also differ in the number of SNPs used in the calculation of each threshold (see Supplementary Table S2). However, even though the BD GWAS was based on the smallest number of subjects and contained the lowest number of genome-wide-associated loci among the three GWAS, the BD PRS showed the strongest associations with BD case status or family membership, underlining the substantial contribution of BD risk variants to the development of BD in the ABiF families.

One limitation of the study is that the subjects of the unrelated control cohort were not systematically screened for psychiatric disorders. The lifetime prevalence of unipolar depression in this cohort (up to 14.4% until the time of the interview) was in line with typically observed numbers [27], the prevalence of BD was not assessed. However, as BD has a lifetime prevalence of ~1%, we expect up to three BD cases among the 277 controls, a number we consider unlikely to have markedly influenced our results. Moreover, using controls unscreened for BD instead of “super-healthy” controls as a comparison to family members and unrelated BD cases represents a conservative approach and thereby strengthens the observed group differences in psychiatric PRS.

Similarly, around one third of the CC_BD_ reported a family history of BD. The CC_BD_ thus do not represent a sample of truly sporadic BD cases. However, the aim of our study was to investigate how members of multiplex BD families differ from typical BD cases regarding the polygenic contribution to their disorder. The observation that ABiF multiplex cases showed a higher polygenic psychiatric risk than CC_BD_, despite part of the CC cases also reporting a family history for BD, thus rather strengthens the validity of our findings.

The present study generated substantial evidence that members of the ABiF families, including unaffected subjects, carried a higher risk burden of common genetic risk variants than an unrelated control sample mainly for the psychiatric disorders BD and SCZ and, at least the FAM_MDD_ cases, for MDD. In line with previous theoretical assumptions [49] and preliminary results from a pilot study in a single ABiF family [26], our results suggest that a high polygenic load of common risk variants is a major contributor to the increased risk for BD and MDD in families with a high density of BD. However, given the modest effect sizes of the PRS, they explained only a fraction of the phenotypic variance, and rare mutations such as copy number variants [50] or rare single-nucleotide variants likely also play an important role in each of the families. Sequencing studies carried out in multiplex families have suggested rare variants are involved in the aetiology of BD [51–53]. To date, however, it has proven difficult to identify replicable causal associations between rare variants and BD susceptibility. In a pilot study that analysed a single ABiF pedigree, we did not identify any rare causal variants for BD [26]. The analysis of rare variants in the remaining ABiF families using next-generation sequencing technologies is envisioned for the future, including integrative analyses in international consortia such as the Bipolar Sequencing Consortium [54]. Of note, the present analyses did not assess single families separately, but integrated PRS associations across all examined 33 ABiF families. Thus, the degree to which common and rare variants shaped the emergence of psychiatric disorders may vary between families.

Furthermore, PRS are commonly based on and applied to sets of unrelated individuals, and polygenic risk might act differently in the case of familial genetic background. Moreover, a broad range of environmental factors have been shown to influence the risk of psychiatric disorders and might act on top of the increased genetic risk in these families. However, environmental factors have not been systematically assessed in the present study. To further enhance our understanding regarding the aetiology of BD, integrated analyses of common and rare variants, as well as of environmental risk in the ABiF families are warranted in the future.

## Supplementary information

Supplemental Material

Supplemental Tables
